# Utilization of Boron Compounds for the Modification of Suberoyl Anilide Hydroxamic Acid as Inhibitor of Histone Deacetylase Class II *Homo sapiens*

**DOI:** 10.1155/2014/104823

**Published:** 2014-08-24

**Authors:** Ridla Bakri, Arli Aditya Parikesit, Cipta Prio Satriyanto, Djati Kerami, Usman Sumo Friend Tambunan

**Affiliations:** ^1^Bioinformatics Group, Department of Chemistry, Faculty of Mathematics and Science, University of Indonesia, Depok 16424, Indonesia; ^2^Mathematics Computation Group, Department of Mathematics, Faculty of Mathematics and Science, University of Indonesia, Depok 16424, Indonesia

## Abstract

Histone deacetylase (HDAC) has a critical function in regulating gene expression. The inhibition of HDAC has developed as an interesting anticancer research area that targets biological processes such as cell cycle, apoptosis, and cell differentiation. In this study, an HDAC inhibitor that is available commercially, suberoyl anilide hydroxamic acid (SAHA), has been modified to improve its efficacy and reduce the side effects of the compound. Hydrophobic cap and zinc-binding group of these compounds were substituted with boron-based compounds, whereas the linker region was substituted with p-aminobenzoic acid. The molecular docking analysis resulted in 8 ligands with Δ*G*_binding_ value more negative than the standards, SAHA and trichostatin A (TSA). That ligands were analyzed based on the nature of QSAR, pharmacological properties, and ADME-Tox. It is conducted to obtain a potent inhibitor of HDAC class II *Homo sapiens*. The screening process result gave one best ligand, Nova2 (513246-99-6), which was then further studied by molecular dynamics simulations.

## 1. Introduction

Cervical cancer is cause by human papillomavirus (HPV) and in the second rank as a cause of cancer death in women worldwide [1]. Cervical cancer occurs in the cervical region, which is located in the hollow area between the vagina and the uterus or commonly called cervix. Cervical cancer can be contagious among all women; a ratio of 1 out of every 4 women is likely to suffer from it [2].

Based on data from the World Health Organization, in 2008, it is estimated 530,232 cases of cervical cancer in the world, with 275,008 mortality cases [3]. Through these data, the estimated global mortality rate from cervical cancer is 50% [2].

HPV is a virus of the family Papillomaviridae and has a nonenveloped, icosahedral-shaped capsid and the double stranded circular DNA as its genetic material [4–6]. It is 7,800–7,900 base pairs long with a 55 nm diameter [7, 8]. HPV has more than 100 different genotypes, and over 40 types of it can infect any part of the epithelial and mucosal lining of the anogenital tissue [9]. The HPV virus is divided into two classes, namely, low-risk HPV (e.g., HPV-6 and HPV-11) and high-risk HPV (e.g., HPV 16 and HPV 18) [10]. Low-risk HPV usually causes a bulge impact on disease areas such as anogenital condylomata (wart) that usually grows on the cervix and vulva [11].

HPV genome is divided into 3 regions, namely, upstream regulatory (URR, noncoding), early gene, and late gene regions [12]. Proteins E6 and E7 oncogenes can make HPV-infected cells to become immortal [13]. E6 protein is associated with ubiquitin (protein ligase), which in turn interacts with p53. It results in the degradation process in the proteosome. E6 also increases the activity of telomerase and induces the creation of immortal cells [14]. E7 protein interacts with the retinoblastoma protein (Rb) and releases the E2F transcription factor that induces expression of genes involved in the process of cell proliferation [15]. E7 oncoprotein can interact directly with the interferon regulatory factor- (IRF-) 1 tumor suppressor proteins that inhibit the performance of the release of E2F and E7, thus increasing the transcriptional activity of cells containing the HPV genome [16]. E6 and E7 activities generally cause epigenetic changes that interfere with the process of cell regulation, apoptosis, DNA-repair processes, hormonal response, and cell differentiation processes, which lead to cervical cancer [17, 18].

Oncoprotein of HPV E6 and E7 in particular has a correlation with the enzyme activity of histone deacetylase (HDAC) [19]. HDAC is a medium for binding with oncogene transcription of genes with the aim of transforming the processes of cells into the media of the viral proliferation [20].

HDAC is an enzyme with EC number 3.5.1 which acts as a catalyst for histone deacetylase [21]. In eukaryotic cells, it is useful for removing acetyl groups from lysine amino acid on a histone tail and wrapping the histones around DNA, thus interfering with the process of gene transcription by binding with transcription factor [22, 23]. In general, there are two regulation processes of gene expression and DNA replication by regulation of chromatin structure [23]. The process of protein acetylation of histone and nonhistone was carried out by the histone acetyl transferases (HATs) and histone deacetylase by histone deacetylase (HDACs) enzyme [24]. These two enzymes are working as opposites because HATs will cause chromatin structure to stretch into euchromatin [25]. It provides space for the specific enzyme or other protein complexes involved in gene expression that serves to increase the activity of transcription and DNA repair [26]. While HDAC causes the release of an acetyl group on the N-acetyl lysine that is available on the histone tail, it causes the DNA to form loops on the histone called heterochromatin [27]. Hence, the transcription of DNA is obstructed and gene expression does not occur properly, thus causing the transformation of normal cells into cancer cells [17, 28]. HDAC inhibition can inhibit the proliferation of epigenetic gene transcription of HPV that causes cancer cells broke down to apoptosis [29].

Suberoyl anilide hydroxamic acid (SAHA) has been through the stages of clinical trials and approved by the U.S. Food and Drug Administration (FDA) as cancer drug [30]. SAHA has the ability to inhibit HDAC, and it could interact with the HDAC metalloenzyme site [31]. Hence, the Zn^2+^ ion lies at the basis of the metalloenzyme site of HDAC [32]. The following is an explanation of each unit in the design of HDAC inhibitors. 

*(1) Zinc Binding Group (ZBG)*. It is a site where a ligand (inhibitor) interacts with Zn^2+^ cofactor contained in the HDAC formed charge relays system with amino acid residues [33]. In general, compounds that can interact with the Zn^2+^ cofactor are nucleophilic compounds, for example, hydroxyl, carbonyl, thiol, carboxylic, and sulfonyl [34]. 

*(2) Linker*. It is the liaison between CAP and ZBG that forms a short-chain hydrocarbons, long-chain hydrocarbons or aromatic such as butane, fatty acids, *γ*-aminobutyric acid (GABA), p-aminobenzoic acid (PABA), furans, and others [35]. The linker is able to interact with amino acid residues found in the cylinder pocket of HDAC enzymes [36]. 

*(3) Hydrophobic Cap (CAP)*. It is a group of compounds that are used to design a cap which is generally a hydrophobic compound that has properties of high lipophilicity [37]. It easily reacts with the surface of the active site and closes the entry point to the enzyme substrate. In general, hydrophobic cap is composed of phenyl, benzyl, furans, polycyclic, and so forth [37, 38].

The reason boron compounds are selected to be substituted at the ZBG and CAP is because the clinical trials have shown that consumption of boron may prevent cervical cancer caused by HPV [37, 39]. By consuming the boron content of 84.1 mg per day, it could prevent cervical cancer [40]. The forms of a functional group of boron compounds that have been proven to have therapeutic effects till date are diazoborin, boronic acid, boronic ester, and benzoxaborole [41]. Carborane has been found to be useful as a good inhibitor and has high lipophilicity properties which are useful for binding with the receptor binding site on the hydrophobic active site of the enzyme [42]. Carborane in the closed form can increase receptor affinity and activity of the enzyme with hydrophobic ligand binding cavity. Therefore, it can inhibit the enzyme activity that contributes to a disruption in the disease [43].

## 2. Materials and Methods

This research method was developed based on established pipeline of our group [44–48]. This is an* in silico* research that involves the use of computerized system. Each query was done using the online and offline software. In this study, MOE 2012.10, ACDLabs, ChemSketch, Toxtree, and VegaZZ were used. Multiple sequence alignment was done in HDAC class II sequences of* Homo sapiens* to obtain the conserved region. Then, the HDAC class II* Homo sapiens* enzyme was computed for its homology modeling with SWISS-MODEL server. As a result, ligand inhibitors for HDAC class II* Homo sapiens* were produced. After both the ligand and the enzyme were ready, the molecular docking simulations were performed. The result of molecular docking simulation was forwarded to the analysis of the existing parameters, namely, pharmacological analysis, ADMET testing, and bioavailability. Further test was carried out to examine the thermodynamic stability of ligand in the presence of solvents with molecular dynamics simulations.

## 3. Results and Discussion

### 3.1. Results

**Figure 1:**
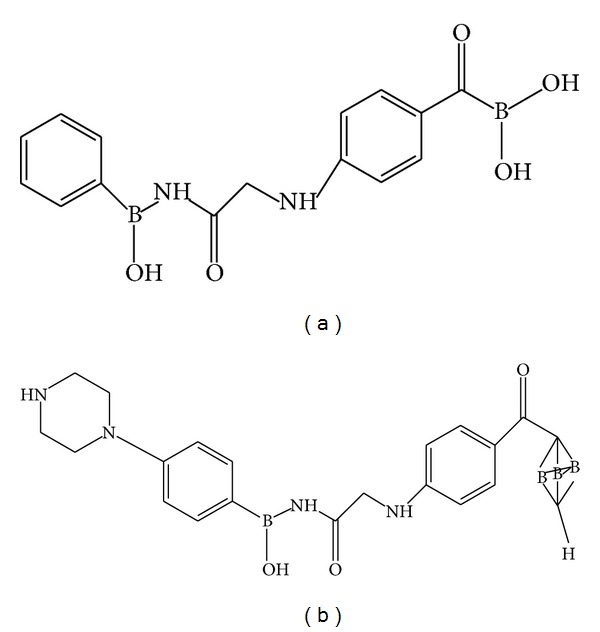
Several examples of ligand modification ((a) and (b)).

The ZBG would be substituted with boronic acid and carborane, part of the linker would be substituted with p-aminobenzoic acid compounds, and hydrophobic parts of cap would be substituted with organoboron compounds from the website of organoborons database (http://www.organoborons.com/) (Figure 1).

The sequences of HDAC class II* Homo sapiens* were searched in a protein sequence database. They could be accessed via the National Center for Biotechnology Information (NCBI). HDAC class II consists of six types of enzymes, namely, HDAC4, HDAC5, HDAC6, HDAC7, HDAC9, and HDAC10. The whole isoform of protein sequences has been encoded in the NCBI Reference Sequence (NCBI RefSeq), GenBank, and UniProt Knowledge Base (UniProtKB)/SWISS-PROT.

After conducting multiple sequence alignment, conserved region sequences were obtained. The obtained sequences of HDAC enzyme code are seen in Table 1. Furthermore, the sequences were piped into the Basic Local similarity Alignment Search Tools (BLAST) which could be accessed through the NCBI website (http://blast.ncbi.nlm.nih.gov/Blast.cgi). BLAST is useful for comparing sequences derived from the conserved region of the existing protein database. The BLAST protein code is written in Table 1.

**Figure 2:**
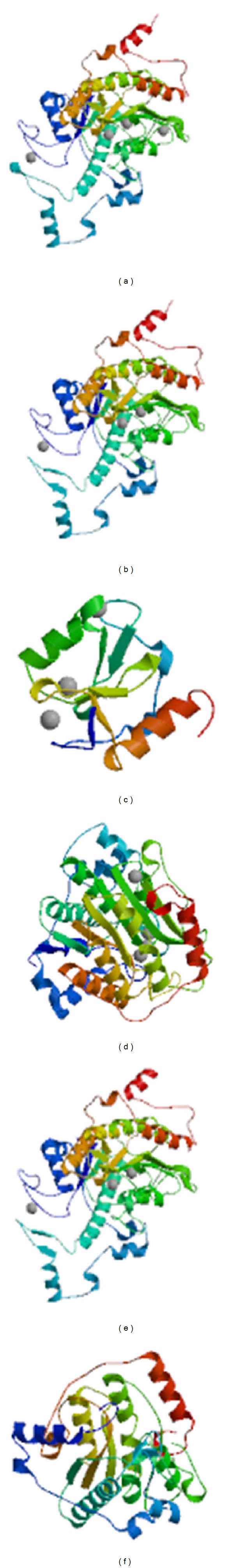
3D Structure of HDAC class II* Homo sapiens*: (a) HDAC4, (b) HDAC5, (c) HDAC6, (d) HDAC7, (e) HDAC9, and (f) HDAC10.

Furthermore, 3D structure modeling was conducted by using SWISS-MODEL server. The predicted 3D image crystal structure of HDAC class II* Homo sapiens* results can be seen in Figures 2(a) to 2(f).

The modeling of the data was also obtained in determining the active site of the enzyme from each HDAC class II* Homo sapiens* enzyme. The active site of each enzyme is listed in Table 2.

Furthermore, molecular docking simulation has been conducted and produced 8 best ligands. The obtained ligands were having Δ*G*_binding_ value lower than SAHA and trichostatin A (TSA) as standards; they are listed in Table 3. The low Δ*G*_binding_ value would facilitate the spontaneous ligand binding reaction with the enzyme to form stable complexes. Deriving from Δ*G*_binding_ value, the inhibition constants of complex formation are in Table 4. The lower the Δ*G*_binding_ value the greater the p*K*_*i*_ value. Docking results also included visualization of the interaction between the ligand with the target enzyme. Ligands would interact with amino acid residues that were owned by the enzyme and also with the active site of the enzyme.  Table 5 presents the interaction between multiple ligands with the enzyme.

Furthermore, the previous modified ligands were forwarded for pharmacological analysis. The analysis was carried out by using Lipinski's rule of five Egan's, and Veber's rules to determine the best drug candidates in its stability and oral bioavailability. According to these rules, the drugs should have a molecular weight of less than 500 Dalton (Da), Log *P* values of less than 5, the number of hydrogen bond donors of less than 5, the number of hydrogen bond acceptors of less than 10, polar surface area of less than 140 Å^2^, and rotation of the ligand binding compound of less than 10 [49, 50]. The test of pharmacological analysis was conducted using FAF-Drugs2 online software.  Table 6 shows the results of pharmacological analysis of each ligand.

As seen in Table 7, the data show that the best 8 ligands have good oral bioavailability with parameters based on the existing rules. Furthermore, an analysis of health impact of ligands has been completed by observing its absorption, distribution, metabolism, excretion, and toxicology (ADMET) properties. The analysis was carried out using Toxtree 2.6.0 software and ACD/I-Lab with the parameter of Benigni-Bossa rule. The method involved analyzing the groups of ligands that have fragments containing substances that cause mutagenic and carcinogenic effects in the cells of the body. Examples of the compounds that could cause these effects are acyl halides, benzyl, esters, epoxides, aliphatic halogen, alkyl nitrites, quinones, hydrazine, polycyclic aromatic hydrocarbons, tiocarbamate, aromatic amines, hydroxylamine, and so forth. Analysis of Toxtree software assessment was generated from data obtained by test using* Salmonella typhimurium* organism or commonly referred to as the AMES test.  Table 8 shows the results of ligands that have been analyzed with the Toxtree software. The result is the best 8 ligands did not have mutagenic and carcinogenic effects.

Furthermore, a probability analysis of ligand's side effects on the human health was conducted. The computational analysis was carried out by using the ACD/I-Lab software and the results for the safest ligands candidate are shown in bold, at Table 9.

**Figure 3:**
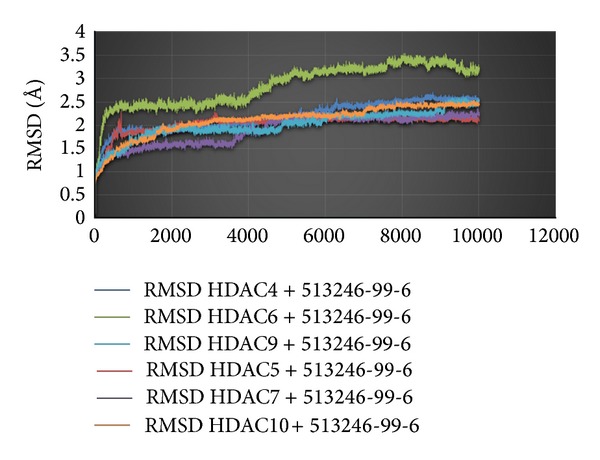
The time (ps) versus RMSD on the molecular dynamics simulation.

Table 9 shows that the entire modified ligand had adverse effects on the gastrointestinal tract, but it was not a problem because in order to distribute the drug, utilization of drug delivery technology could be in place to target the receptor. After passing the test, it was determined that the best ligand is Nova2 (513246-99-6). This is because of lower Δ*G*_binding_ value than the standard. Its value is almost the same in all of the HDAC class II enzymes. The best ligands were tested using molecular dynamics simulations to look at their stability due to the changes of solvent as well as temperature. The process was divided into three phases with the first phase of initialization temperature of 300 K, equilibration stage and heating temperature of 310 K, and the last stage of production. The simulated stage happened when the drug met the solvent and the occurrence of temperature changed and when the drug was distributed and reached its intended target. The dynamic simulation result is shown in Figure 3 as RMSD versus time (ps) curve at the molecular dynamic stage of the best drug candidate, Nova2 (513246-996), against the target enzyme.

The graph shows that Nova2 ligand (513246-99-6) was stable at the time of 5000 ps. However, progression in HDAC6 shows an increase in the curve due to the shallowness of the binding pocket; the ligand was somewhat less stable due to the influence of the solvent, which was outside the surface of the enzyme.

### 3.2. Discussion

Due to the versatility of organoboron compounds, they have proved themselves to be useful at the field or chemical science [51]. The synthesizing of organoboron compounds has been proven to be physically possible [52]. The flexibility of organoboron as electrophile and nucleophile compound adds a tunable property in the drug design [51]. The efficacy of organoboron compounds has been proven in the fungicide and bactericide-based in vitro experiment [53]. More specifically, the closo-carborane compounds have indeed become a logical choice for drug design due to their biological activity [43]. The hydrophobic property of closo-carborane is making chemical modifications on various compounds feasible, as a measure to observe its pharmacochemistry properties [54]. Hence, Velcade © is the only boron-based therapeutic in the market that is useful to threat multiple myolema, and there are several more that undergo clinical trials, including Talabostat © as lung cancer drug candidate [41, 55]. In this end, it is expected that there will be more boron based drugs in the market.

The computational measure for docking of simple boronic acid based compounds was already utilized and has paved the way for serious organoboron based rational drug design [56]. The starting point of the development of our pipeline was the optimization of docking method, which would be improved later on [57]. Our experience in HPV drug design was based on design of organic compounds as drug candidates [46, 58]. However, as carbon and boron based compounds have some similarity of physicochemical properties, the utilization of boron as carbon substitute for drug candidate becomes more feasible [59]. Now, closo-carborane and boronic acid based lead compounds were successfully designed based on our established methodology. The low toxicity and degradability of boronic acid into safer boric acid made it an environmental friendly compound [60]. It is also observed that closo-carborane could improve the hydrophobic interaction with enzyme [43]. Due to the robustness of organoboron compounds, the existing pipeline could be applied “as it is” without any major modification, for working with organometalloid compounds. Moreover, the designed organoboron compounds are still below the threshold of Lipinski's rule molecular weight barrier, so it will be much simpler to develop them [59]. Thus, the Nova2 (513246-99-6) that was a combined compound from SAHA and (4-piperazin-1-ylphenyl) boronic acid was chosen as the most feasible drug candidate [61]. The slightly acidic properties of the boronic acid functional groups, combined with the electronegativity tendency from its nitrogen atom, may contribute to the inhibition activity to the Zn^2+^ metalloenzyme pocket [62]. However, the acidic property of Nova2 (513246-99-6) should be taken into account for its oral delivery measure, as it could inflict certain hazard for heartburn patient. In this end, prodrug construction should be considered in its synthesis strategy [63].

The wet laboratory experiment that is working with the interaction of protein and organoboron compound was already proved to be feasible [64]. Moreover, organoboron compound is just starting to be applied as radiotherapy agent [65]. The synthesis pathway for both boronic acid and closo-carborane derivatives are already applied by some research group [66, 67]. Thus, in order to soften the complexity of the synthesis, a prediction method will be utilized to evaluate the synthesis accessibility [68–70]. To this end, by applying the information from in silico results, it is expected that the laboratory synthesis and bioassay experimentation for organoboron compound should be straightforward and not difficult.

## 4. Conclusion

Modification of ZBG and CAP at SAHA with a boron compound and the p-aminobenzoic (PABA) as linker turns out to yield results as expected, as they show better HDAC inhibition than SAHA. This modification resulted in a total of 1,100 ligands. After going through molecular docking simulations, the top 8 ligands were obtained; they have much lower Δ*G*_binding_ than standard SAHA and TSA. The ligands were Nova2 (9058064-6), Nova2 (95752-88-8), Nova2 (88765-82-6), Nova2 (unique10), Nova2 (16876-27-0), Nova2 (513246-99-6), Nova2 (unique80), and Nova2 (279262-23-6). After undergoing the screening process of QSAR, pharmacological properties, and ADME-Tox, 4 best ligands were collected that can be used as drug candidates. They were Nova2 (95752-88-8), Nova2 (16876-27-0), Nova2 (513246-99-6), and Nova2 (279262-23-6). After a thorough analysis, Nova2 (513246-99-6) was concluded as the best drug candidate, and it was piped to the molecular dynamics simulation process.

## Figures and Tables

**Table 1:** The sequence code for each HDAC enzyme.

Enzyme	Sequence code
HDAC4	P56524.3
HDAC5	Q9UQL6.2
HDAC6	Q9UBN7.2
HDAC7	Q8WUI4.2
HDAC9	Q9UKV0.2

**Table 2:** Catalytic site of HDAC class II *Homo sapiens *enzyme.

Enzyme	Active site
HDAC4	Zn^2+^ cofactor with Asp196, His198, and Asp290 residue
HDAC5	Zn^2+^ cofactor with Asp870, His872, and Asp964
HDAC6	Zn^2+^ cofactor with Cys5, His7, and Cys78
HDAC7	Zn^2+^ cofactor with Asp707, His709, and Asp801
HDAC9	Zn^2+^ cofactor with Asp820, His822, and Asp914
HDAC10	Zn^2+^ cofactor with Asp172, His174, and Asp265

**Table 3:** The Δ*G*_binding_ data result of the best ligands binding toward HDAC class II *Homo sapiens*.

Ligand	Gibbs free energy (Δ*G*_binding_) towards HDAC class II *Homo sapiens *					
	HDAC4	HDAC5	HDAC6	HDAC7	HDAC9	HDAC10
Nova2 (9058064-6)	**−25,83**	−28,94	**−29,43**	**−31,33**	−25,24	−24,01
Nova2 (95752-88-8)	−18,25	**−31,67**	−28,87	−28,14	**−26,74**	**−25,96**
Nova2 (88765-82-6)	−23,63	−29,22	−28,20	−25,41	−24,20	−23,32
Nova2 (Unique10)	−23,79	−29,14	−27,58	−26,66	−24,39	−20,90
Nova2 (16876-27-0)	−16,96	−27,72	−25,88	−24,31	−24,80	−22,84
Nova2 (513246-99-6)	−22,69	−28,57	−27,01	−26,72	−24,92	−21,75
Nova2 (Unique80)	−22,14	−25,00	−26,80	−24,51	−23,43	−20,83
Nova2 (279262-23-6)	−22,13	−27,46	−26,04	−27,09	−24,25	−20,68
SAHA	−13,93	−17,28	−15,35	−14,54	−16,19	−15,37
TSA	−14,65	−15,21	−14,69	−13,50	−14,28	−13,01

Note: numbers in bold show the lowest binding energy.

**Table 4:** The inhibition constant (p*K*_*i*_) of ligand binding with HDAC class II *Homo sapiens*.

Ligand	Inhibition constant (p*K*_*i*_)					
	HDAC4	HDAC5	HDAC6	HDAC7	HDAC9	HDAC10
Nova2 (9058064-6)	18,82	21,09	21,44	22,82	18,39	17,49
Nova2 (95752-88-8)	13,30	23,07	21,03	20,50	19,48	18,92
Nova2 (88765-82-6)	17,21	21,29	20,54	18,51	17,63	16,99
Nova2 (Unique10)	17,33	21,23	20,09	19,42	17,77	15,23
Nova2 (16876-27-0)	12,36	20,20	18,86	17,71	18,07	16,64
Nova2 (513246-99-6)	16,53	20,81	19,68	19,47	18,15	15,85
Nova2 (Unique80)	16,13	18,21	19,53	17,86	17,07	15,18
Nova2 (279262-23-6)	16,13	20,01	18,97	19,73	17,67	15,07
SAHA	10,15	12,59	11,19	10,59	11,80	11,20
TSA	10,68	11,08	10,70	9,84	10,40	9,48

**Table 5:** Ligand interaction with amino acid residues of HDAC class II *Homo sapiens*.

Ligand	Docking interactions					
	HDAC4	HDAC5	HDAC6	HDAC7	HDAC9	HDAC10
Nova2 (9058064-6)	Zn^2+^, Arg37, Trp195, Asp220	Zn^2+^, His833, His872, Trp869, Leu892, Tyr937, Phe983	Zn^2+^, His7, Leu8, Ala10	Zn^2+^, Arg547, His843, Asp801, His709, Arg731, Trp706	Zn^2+^, Arg606, Asp820, Tyr887, Ala911, Gly912, Asp914	Zn^2+^, Arg30, Trp171, His174, Arg196, Phe254, Asp265

Nova2 (95752-88-8)	Zn^2+^, Arg37	Zn^2+^, His833, Asp870, His872, Tyr937, Trp869, Asp964	Zn^2+^, His7, Val9	Zn^2+^, Arg547, Tyr774, Gly799, Asp801, His843	Zn^2+^, Arg660, Phe833, Gly912, Asp914	Zn^2+^, Arg30, Trp171, Gly263, Phe282, Gly303, Gly304

Nova2 (88765-82-6)	Zn^2+^, Asp290, Glu329, Trp195, Phe289	Zn^2+^, His872, Arg710, Asp870, Asp964, Gly1005	Zn^2+^, Arg 47, Ala80	Zn^2+^, Arg547, Ala798, Phe800, Gly841, His843,	Zn^2+^, Asp820, Leu842, Glu853, Asp914	Zn^2+^, Asp172, Asp265

Nova2 (Unique10)	Zn^2+^, Arg37, trp195, Ser287, Gly288	Zn^2+^, His833, His872, Asp964, Leu973	Zn^2+^	Zn^2+^, Arg547, Asp707, Trp706, Asp801, Gly799, His843,	Zn^2+^, Arg660, Gly791, Asp914	Zn^2+^, Asp172, Tyr238, Asp265, Glu302

Nova2 (16876-27-0)	Zn^2+^, Gly288	Zn^2+^, Asp870, His872, Arg710	Zn^2+^	Zn^2+^, Arg547	Zn^2+^, Arg660, Asp914	Zn^2+^, Asp172, Asp265

Nova2 (513246-99-6)	Zn^2+^, Ser287, Gly288, Asp290, Leu304	Zn^2+^, His833, Asp870, His872, Gly962	Zn^2+^, Ser72, Trp74, Tyr77	Zn^2+^, His709, Gly799, Asp801, His843	Zn^2+^, Arg660, Arg778, His822, Asp914, His956	Zn^2+^, Trp171, Asp172, Tyr238, ASp265, Ser266

Nova2 (Unique80)	Zn^2+^, Gly288, Asp290	Zn^2+^, Arg710, Trp869, Ala961, Phe963	Zn^2+^, Leu64, Typ74	Zn^2+^, Val708, Gly812, His843	Zn^2+^, His822, Asp914, Gly 954	Zn^2+^, Trp171, Asp172, Trp228, Tyr238, Gly303

Nova2 (279262-23-6)	Zn^2+^, Ser287, Asp290, Phe209, Ala327	Zn^2+^, Arg710, Asp870, Gly962, Gly1005	Zn^2+^, Val9	Zn^2+^, Arg547, Trp706, Asp801, His843	Zn^2+^, Arg660, Asp820, Asp914	Zn^2+^, Asp172, Tyr238, Phe264, Asp265

SAHA	Zn^2+^, Asp196, His198, Asp290	Zn^2+^, Asp870, His872, Asp964, Gly1005	Zn^2+^, Cys5, His7, Cys75, Cys78	Zn^2+^, Asp707, His709, Asp801, Tyr813	Zn^2+^, Arg660, Gly954, Gly955	Zn^2+^, Trp171, Asp172, Val173, His174, Asp265, Phe282

TSA	Zn^2+^, Asp196, His198, Asp290, Ser287	Zn^2+^, Asp870, His872, Asp964, Arg710	Zn^2+^, Cys75, Cys78	Zn^2+^, Asp707, His709, Asp801	Zn^2+^, Asp820, His822, Asp914, Gly955	Zn^2+^, Asp172, Val173, His174, Asp265, Phe282

**Table 6:** Ligands screening using Lipinski's rule.

Ligand	Mw	log⁡P	TPSA	Rot. bond	HBD	HBA	Violation
Nova2 (9058064-6)	425,63	−1,52	141,75	10	6	8	1
Nova2 (95752-88-8)	465,74	2,40	88,69	9	3	7	0
Nova2 (88765-82-6)	423,66	1,26	102,68	9	4	7	0
Nova2 (Unique10)	423,70	2,44	90,46	12	4	6	0
Nova2 (16876-27-0)	409,63	1,12	81,79	8	2	7	0
Nova2 (513246-99-6)	425,70	1,28	93,70	8	4	7	0
Nova2 (Unique80)	471,79	2,50	79,90	9	2	7	0
Nova2 (279262-23-6)	437,69	1,28	90,90	9	3	7	0
SAHA	264,32	2,47	78,42	8	3	5	0
TSA	302,37	2,68	69,63	6	2	5	0

Note: Mw: molecular weight; log⁡P: octanol-water partition coefficient; TPSA: topological polar surface area; Rot. bond: the amount of rotatable bond; HBD: the amount of hydrogen donor atom; HBA: the amount of hydrogen atom acceptor; violation: the amount of the violation to Lipinski's rule.

**Table 7:** Ligand bioavailability.

Ligand	Egan's rule	Veber's rule	Bioavability	
			% *F*(Oral) > 30%	% *F*(Oral) > 70%
Nova2 (9058064-6)	Good	Low	0.03	0.01
Nova2 (95752-88-8)	Good	Good	0.85	0.48
Nova2 (88765-82-6)	Good	Good	0.85	0.27
Nova2 (Unique10)	Good	Good	0.76	0.27
Nova2 (16876-27-0)	Good	Good	0.85	0.48
Nova2 (513246-99-6)	Good	Good	0.76	0.27
Nova2 (Unique80)	Good	Good	0.76	0.23
Nova2 (279262-23-6)	Good	Good	0.59	0.27

**Table 8:** Carcinogenicity and mutagenicity of the best ligands.

Ligand	Potential carcinogen based QSAR	Genotoxic carcinogenicity	Nongenotoxic carcinogenicity	Potential *S. typhimurium* mutagenicity
Nova2 (9058064-6)	No	Negative	Negative	No
Nova2 (95752-88-8)	No	Negative	Negative	No
Nova2 (88765-82-6)	No	Negative	Negative	No
Nova2 (Unique10)	No	Negative	Negative	No
Nova2 (16876-27-0)	No	Negative	Negative	No
Nova2 (513246-99-6)	No	Negative	Negative	No
Nova2 (Unique80)	No	Negative	Negative	No
Nova2 (279262-23-6)	No	Negative	Negative	No
SAHA	No	Negative	Negative	No
TSA	No	Positive	Negative	Yes

**Table 9:** The probability of side effects of ligands on human health.

Ligand	The probability of side effects (0 < *x* < 1)					
	Blood	Cardiovascular system	Gastrointestinal system	Kidney	Liver	Lung
Nova2 (9058064-6)	0.24	0.07	0.81	0.10	0.08	0.62
Nova2 (95752-88-8)	0.24	0.07	0.81	0.10	0.08	0.62
Nova2 (88765-82-6)	0.24	0.07	0.81	0.10	0.08	0.62
Nova2 (Unique10)	0.24	0.07	0.81	0.10	0.08	0.62
Nova2 (16876-27-0)	0.24	0.07	0.81	0.10	0.08	0.62
**Nova2 (513246-99-6)**	0.24	0.07	0.81	0.10	0.08	0.62
Nova2 (Unique80)	0.24	0.07	0.81	0.10	0.08	0.62
Nova2 (279262-23-6)	0.24	0.07	0.81	0.10	0.08	0.62
SAHA	0.36	0.25	0.07	0.11	0.11	0.37
TSA	0.59	0.50	0.51	0.27	0.52	0.63
